# Effect of early intubation on patient-centered outcomes in urosepsis: a retrospective multicenter cohort study

**DOI:** 10.1186/s40560-025-00829-4

**Published:** 2025-10-23

**Authors:** Masafumi Suga, Ryan Ling, Sho Katsuragawa, Yahya Shehabi, David Pilcher, Ashwin Subramaniam

**Affiliations:** 1 Department of Surgery Monash Health, School of Clinical Sciences at Monash Health, Monash University, Clayton, VIC Australia; 2 Department of Intensive Care, Monash Medical Centre, Monash Health, Clayton, VIC Australia; 3 Department of Emergency and Critical Care Medicine, Hyogo Emergency Medical Centre, Hyogo, Japan; 4 Yong Loo Lin School of Medicine, National University of Singapore, National University Health System, Singapore, Singapore; 5 Australian and New Zealand Intensive Care Research Centre (ANZIC-RC), Department of Epidemiology and Preventive Medicine, Monash University, Melbourne, VIC Australia; 6 Department of Anaesthesia, Khoo Teck Puat Hospital, National Healthcare Group, Singapore, Singapore; 7 Centre for Endocrinology and Reproductive Health, Hudson Institute of Medical Research, Clayton, VIC Australia; 8 Department of Intensive Care, Victorian Heart Hospital, Monash Health, Clayton, VIC Australia; 9 Department of Intensive Care, Alfred Health, Melbourne, VIC Australia; 10 Centre for Outcome and Resource Evaluation, Australian and New Zealand Intensive Care Society, Melbourne, VIC Australia; 11 Department of Intensive Care, Dandenong Hospital, Monash Health, Dandenong, VIC Australia; 12 Peninsula Clinical School, Monash University, Frankston, VIC Australia; 13 Department of Intensive Care, Epworth Healthcare, Richmond, VIC Australia

**Keywords:** Urosepsis, ANZICS adult patient database, Early intubation, ICU outcomes, Hospital mortality, Mechanical ventilation

## Abstract

**Background:**

Urosepsis has a reported mortality rate of up to 13.5%, and approximately 38% of affected patients require intubation. This study evaluated the association between the timing of intubation and in-hospital mortality among patients with urosepsis.

**Methods:**

We conducted a multicenter retrospective cohort study using the Australian and New Zealand Intensive Care Registry Adult Patient Database. Adult ICU patients (≥ 16 years) with a primary diagnosis of urosepsis admitted between 1 January 2018 and 1 April 2023 were included. Patients were classified into early (≤ 24 h from ICU admission) or delayed (> 24 h) intubation groups. The primary outcome was in-hospital mortality. Secondary outcomes included ICU and hospital lengths of stay (LOS), mortality at 6, and 12 months. Outcomes were analyzed using multivariable logistic or linear regression models.

**Results:**

Of 1,235 patients across 151 sites, 983 patients (79.6%) received early intubation. In-hospital mortality was similar between early and delayed intubation groups (19.2% vs. 17.5%, p = 0.52). Early intubation was not associated with in-hospital mortality (adjusted odds ratio [OR] = 0.76; 95% confidence intervals [95% CI] 0.51–1.13). Patients with early intubation had shorter ICU LOS (adjusted point estimate = −2.94 days; 95% CI −3.90 to −1.98) but not hospital LOS. There was no association between early intubation and mortality at 6 months (adjusted OR = 0.76; 95% CI 0.53–1.10) and 12 months (adjusted OR = 0.75; 95% CI 0.53–1.06).

**Conclusions:**

Early intubation within the first 24 h after ICU admission was not associated with reduced in-hospital or long-term mortality among patients with urosepsis.

*Trial registration*: Alfred Hospital Ethics Committee (Reference 762/24) and the Australian and New Zealand Intensive Care Society (ANZICS) Centre for Outcome and Resource Evaluation Management Committee.

**Supplementary Information:**

The online version contains supplementary material available at 10.1186/s40560-025-00829-4.

## Background

While the overall incidence of sepsis has declined, there has been a notable rise in severe urinary tract infections and urosepsis [1], with correspondingly poor outcomes and complex management requirements [2], including intubation and mechanical ventilation (MV). In patients receiving MV, the timing of intubation could remain an important decision in the management of urosepsis. In general sepsis, intubation is typically indicated in patients with severe hypoxemia, altered mental status, or signs of respiratory fatigue. In contrast, in urosepsis, there are often no or only minor indications for intubation aside from circulatory shock, which can make it challenging for clinicians to determine the appropriate timing for initiating mechanical ventilation. In the context of sepsis, the evidence regarding the timing of intubation and initiation of mechanical ventilation remains equivocal [3–7]. While some [8] have shown that patients with delayed intubation had fewer days alive without organ support at day 28, others have demonstrated that intubation within 24 h after intensive care unit (ICU) admission was not associated with hospital mortality but resulted in fewer 28-day hospital-free days [5]. Whether early intubation affects hospital mortality also remains uncertain [6, 7, 9]. While there may be potential benefits of early intubation in sepsis, its optimal timing remains controversial and warrants further studies.

Restricting the cohort to urosepsis improves homogeneity by minimizing variability in infection source; however, it may also bias the population toward less severe cases compared with other sepsis syndromes, thereby limiting generalizability. To date, no studies have specifically investigated the association of early intubation in patients with urosepsis, nor have any comprehensively analyzed patient subgroups in the existing literature. In the context of urosepsis, early intubation may theoretically offer benefits of reducing the physiological stress associated with severe infection [10]. However, the timing of intubation must be carefully balanced against the harms of prolonged MV, such as ventilator-associated events [11], barotrauma [12], and potentially longer ICU stays [13]. Currently, there are no clear guidelines regarding the timing of tracheal intubation and initiation of MV [14]. To address this gap, in this registry-based study, we aimed to evaluate the association between early intubation and MV and mortality in patients with urosepsis admitted to ICUs.

## Methods

### Study design and participants

In this multicenter retrospective registry-based cohort study, we included all adults (≥ 16 years) admitted to an ICU between 1st January 2018 and 1st April 2023 with a non-operative ICU admission diagnosis of urosepsis based on the Australian and New Zealand Intensive Care Society (ANZICS) modification of the Acute Physiological and Chronic Health Evaluation (APACHE) IV codes (502 and 504). We excluded patients who were transferred from another ICU, admitted for palliative and/or organ donation purposes, not intubated, or had missing data on in-hospital mortality, intubation status, or survival time. ICU readmissions during the same hospitalization were also excluded; only the initial ICU admission was included.

### Data sources, definition, and collection

Data were extracted from the ANZICS-APD (Adult Patient Database), a bi-national clinical quality registry dataset that collects de-identified information on all admissions to contributing adult ICUs in Australia and New Zealand. The ANZICS-APD captures admissions to 98% of ICUs in Australia and 67% of ICUs in New Zealand. Data collectors receive regular training and quality assurance reviews, and data are collected using a standardized data dictionary [15]. In addition, regular automated data checks further ensure the validity of recorded data. The registry captures demographic, diagnostic, biochemical and physiological data from the first 24 h of ICU admission, interventions, and outcomes at hospital discharge. ICU admission records were linked to the date of death recorded in the national death index register from 1st January 2018 through 1st April 2023 using an encoded linkage key. We extracted data on patient age, sex, Indigenous status, comorbidities (as defined for the APACHE II, III and IV scoring systems), frailty status (clinical frailty scale; CFS), ICU admission source, admission diagnosis, acute illness severity (using the sequential organ failure assessment [SOFA] score or Australia New Zealand Risk of Death [ANZROD] mortality prediction model), ICU organ supports (mechanical ventilation, non-invasive ventilation, vasopressors, extracorporeal membrane oxygenation [ECMO], renal replacement therapy), treatment limitation at admission to ICU, ICU and hospital mortality, ICU and hospital length of stay, and discharge destinations. Post-discharge mortality was obtained through linkages to the national death registries of Australia and New Zealand.

Early intubation was defined as intubation and initiation of MV within the first 24 h after ICU admission based on the APD database, while delayed intubation was defined as intubation and MV occurring more than 24 h after ICU admission.

### Outcomes

The primary outcome was in-hospital mortality. Secondary outcomes included mortality at 3, 6, and12 months of follow-up, ICU and hospital length of stays, duration of MV, need for tracheostomy, ICU complications (delirium and pressure injury), and non-home discharge destination at hospital discharge.

### Data analysis

We performed data analysis using R4.4.1 (The R Foundation, Boston, MA, USA) and STATA17 (Stata Corp. College Station, Texas, USA). We used a two-sided p < 0.05 to indicate statistical significance. Categorical data are presented as counts and percentages, and continuous data as mean (standard deviation [SD]) or median (interquartile range [IQR]), depending on the distribution. We compared between groups using the Chi-squared, Student t-test, or log-rank tests as appropriate. Survival probability was estimated using the Kaplan–Meier method.

We analyzed the association between early intubation and mortality outcomes, including in-hospital, 3-month, 6-month, and 12-month mortality, using multivariable logistic regression. Results were presented as odds ratios (OR) with corresponding 95% confidence intervals (95%CI), adjusted for baseline covariates identified by a directed acyclic graph (DAG; Supplementary Fig. 1). These covariates included age, SOFA score, CFS categories (non-frail [CFS 1–4], frail [CFS 5–8], and unknown frailty), hospital admission source (other hospital without an emergency department [ED], other healthcare facilities, rehabilitation, or other hospital via ED), treatment limitations at ICU admission, and emergency response admission.

As secondary outcomes, we analyzed ICU and hospital length of stay using multivariable linear regression, reporting adjusted point estimates with corresponding 95%CI, using the same baseline covariates as in the multivariable logistic regression. The other secondary outcomes were presented descriptively. ORs of the primary and secondary outcomes were also displayed in a forest plot.

We conducted several sensitivity analyses. First, we repeated the primary multivariable analyses using the APACHE III score as an alternative measure of illness severity. Unlike the SOFA score, which relies solely on the PaO_2_/FiO_2_ ratio for respiratory assessment, APACHE III incorporates multiple respiratory parameters and is therefore less susceptible to the influence of treatment decisions such as intubation and MV. The same covariates were used, with the SOFA score and age replaced by the APACHE III score. Second, we conducted a sensitivity analysis using individual components of the APACHE III score, excluding the Glasgow coma scale (GCS) and respiratory-related components (respiratory rate and oxygenation variables), which may potentially influence treatment decisions. Third, we conducted a post hoc sensitivity analysis excluding patients discharged within the first 24 h of ICU admission, to address potential immortal time bias.

Predefined subgroups to investigate the primary outcome included biological sex, age categories (< 65 vs. ≥ 65 years), frailty status (non-frail vs. frail), shock status (with vs. without vasopressor) and admission source (ED vs. general ward).

## Results

### Baseline characteristics

**Fig. 1:**
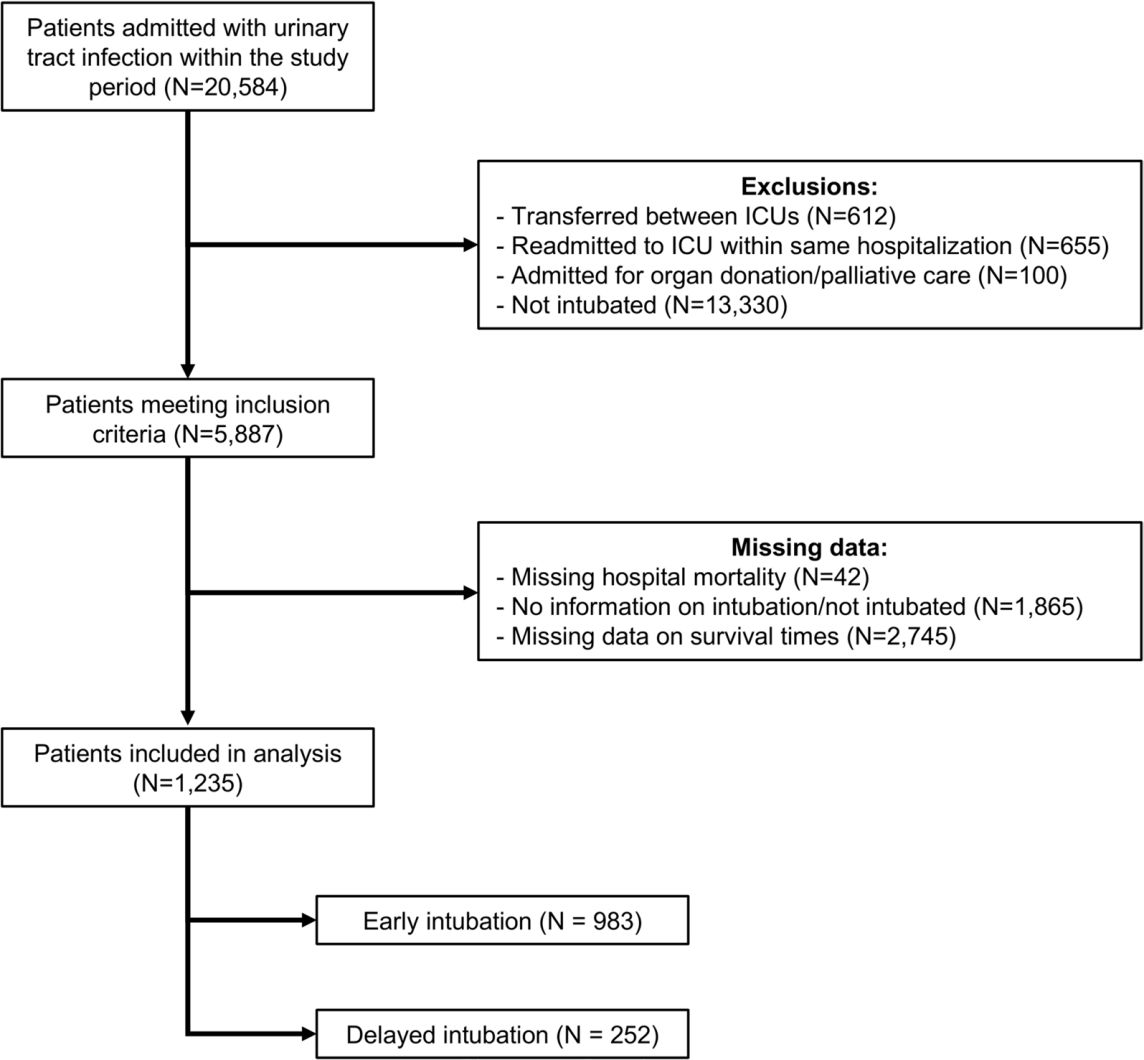
Flow diagram of patient inclusion criteria

During the study period, a total of 20,584 patients from 151 sites with urosepsis were admitted to the ICU. Of these, 13,330 patients (64.8%) who were not intubated during their hospital stay were excluded. At baseline, patients who were not intubated had substantially lower illness severity compared to those who underwent intubation, as reflected by markedly lower APACHE II, APACHE III, ANZROD, and SOFA scores (Supplementary Table 1). After applying the inclusion and exclusion criteria, 1,235 patients were included in the final analysis; 983 patients in the early intubation group and 252 patients in the delayed intubation group were included in the final analysis (Fig. 1).

Baseline characteristics are presented in Table 1. The median age of the enrolled patients was 67.8 (IQR: 55.8–76.0) years and 53.4% (n = 659) were women. The median PaO_2_/FiO_2_ ratio (worst within 24 h) was 215.0 (IQR: 144.9–310.0), and the median GCS was 14 (IQR: 11–15). Initial SOFA score, APACHE-II score, APACHE-III score, and ANZROD score were higher in the early intubation group than in the delayed intubation group. PaO_2_/FiO_2_ ratio and GCS were lower in the early intubation group. Septic shock, defined as vasopressor requirement in combination with a serum lactate > 2 mmol/L [16], was more frequent in the early intubation group than in the delayed intubation group.

**Table 1:** Patient characteristics

Demographic variable	Early intubation (n = 983)	Delayed intubation (n = 252)	Total (n = 1235)	P-value
Age (years)	68.2 [56.8–76.0]	66.8 [53.9–75.6]	67.8 [55.8–76]	0.59
Age ≥ 65 years	581 (59.1)	137 (54.4)	718 (58.1)	0.17
Female sex	514 (52.3)	145 (57.5)	659 (53.4)	0.47
BMI (kg.m^−2^)*	29.4 [25.4–35.6]	29.9 [24.7–35.4]	29.4 [25.3–35.6]	0.51
Indigenous status^†^	60 (6.1)	13 (5.2)	73 (5.9)	0.80
Hospital classification				
Rural/Regional	137 (13.9)	38 (15.1)	175 (14.2)	0.59
Metropolitan	248 (25.2)	70 (27.8)	318 (25.7)	
Tertiary/Teaching	527 (53.6)	123 (48.8)	650 (52.6)	
Private	71 (7.2)	21 (8.3)	92 (7.4)	
Hospital admission source				
Home/Hospital in the home	708 (72.0)	189 (75.0)	897 (72.6)	0.12
Other acute hospital (not ICU/ED)	100 (10.2)	30 (11.9)	130 (10.5)	
Nursing home/Chronic care	29 (3.0)	8 (3.2)	37 (3.0)	
Rehabilitation	3 (0.3)	2 (0.8)	5 (0.4)	
Other hospital—ED	143 (14.5)	23 (9.1)	166 (13.4)	
ICU admission source				
Via ED	728 (74.1)	168 (66.7)	896 (72.6)	0.02
Via general ward	255 (25.9)	84 (33.3)	339 (27.4)	
Emergency response admission	196 (19.9)	61 (24.2)	257 (20.8)	0.27
Comorbidities				
Chronic respiratory condition	78 (7.9)	22 (8.7)	100 (8.1)	0.68
Chronic cardiovascular condition	91 (9.3)	25 (9.9)	116 (9.4)	0.75
Chronic renal failure	58 (5.9)	14 (5.6)	72 (5.8)	0.84
Chronic liver disease	27 (2.7)	9 (3.6)	36 (2.9)	0.49
Diabetes mellitus	342 (40.4)	97 (47.1)	439 (35.5)	0.08
Immunosuppressive therapy	66 (6.7)	24 (9.5)	90 (7.3)	0.13
Metastatic cancer	32 (3.3)	11 (4.4)	43 (3.5)	0.39
Lymphoma	12(1.2)	3 (1.2)	15 (1.2)	> 0.99
Leukemia	10 (1.0)	3 (1.2)	13 (1.1)	0.74
APACHE II score	26.0 [21.0–31.0]	23.0 [17.0–28.0]	25.0 [20.0–31.0]	< 0.001
APACHE III score	84.0 [65.0–105.0]	74.0 [60.0–91.0]	81.0 [64.0–102.0]	< 0.001
ANZROD predicted mortality (%)	22.9 (22.9)	13.8 (16.6)	21.0 (22.1)	< 0.001
SOFA score	8.0 [5.0–10.0]	6.0 [4.0–8.0]	7.0 [5.0–10.0]	< 0.001
CFS categories				
CFS 1–4, non-frail	441 (44.9)	106 (42.1)	547 (44.3)	0.61
CFS 5–8, frail	282 (28.7)	80 (31.7)	362 (29.3)	
CFS unknown	260 (26.4)	66 (26.2)	326 (26.4)	
Treatment limitation at ICU admission	121 (12.3)	25 (9.9)	146 (11.8)	0.30
Septic shock^§^	470 (47.8)	92 (36.5)	562 (45.5)	0.001
Organ supports during ICU stay				
Non-invasive ventilation	184 (18.7)	69 (27.4)	253 (20.5)	< 0.001
Vasopressors	796 (81.0)	188 (74.6)	984 (79.7)	< 0.001
ECMO	1 (0.1)	2 (0.8)	3 (0.2)	< 0.001
Renal replacement therapy	248 (25.2)	63 (25.0)	311 (25.2)	0.003
Total bilirubin (mg/dL)	16 [10–28]	15 [9.0–29.0]	16 [9–28]	0.51
Platelets (10^3^/μL)	163 [108–229]	147 [81–220]	151 [85–220]	0.033
Potassium (mEq/L)	4.6 [4.2–5.1]	4.4 [4.1–4.9]	4.5 [4.2–5.1]	0.004
Creatinine (μmol/L)	192.5 [120.0–294.0]	170.0 [109.0–280.0]	190.0 [116.0–293.0]	0.22
Lactate (mmol/L)	2.8 [1.6–5.4]	2.2 [1.2–3.8]	2.6 [1.5–5.0]	< 0.001
Vital signs (worst within 24 h)				
P/F ratio	204.0 [138.0–300.0]	250.0 [175.0–343.5]	215.0 [144.7–310.0]	< 0.001
P/F ratio < 200	455 (49.0)	78 (34.4)	533 (43.2)	< 0.001
GCS	14.0 [11.0–15.0]	15.0 [13.0–15.0]	14.0 [11.0–15.0]	< 0.001
MAP (mmHg)	61.0 [55.0–67.0]	61.0 [55.0–66.0]	61 [55–66]	0.86
24 h urine output (ml)	1,312 [570–2,198]	1,550 [736–2,336]	1354 [598–2218.5]	0.44

Data are n (%), mean (SD), or median [IQR]

*BMI* body mass index, *ICU* intensive care unit, *ED* emergency department, *APACHE* Acute Physiology and Chronic Health Evaluation, *ANZROD* Australia New Zealand Risk of death, *SOFA* Sequential Organ Failure Assessment, *CFS* clinical frailty scale, *ECMO* extracorporeal membrane oxygenation, *P/F ratio* PaO_2_/FiO_2_ ratio, *GCS* Glasgow coma scale, *MAP* mean arterial pressure, *IQR* inter-quartile range, *SD* standard deviation

All between-group comparisons were statistically significant (P < 0.05), except for age, sex, obesity (BMI), indigenous status, hospital classification, admission source, emergency response admission, comorbidities, total bilirubin, creatinine, MAP, and 24 h urine output

^*^BMI: We had data available only on height or weight

^†^Indigenous: the patient identifies as indigenous to the country where they receive treatment. In Australia, a patient who identifies as Aboriginal or Torres Strait Islander should be coded as indigenous

^§^Septic shock: defined as vasopressor requirement in combination with a serum lactate > 2 mmol/L

### Primary outcome

**Fig. 2:**
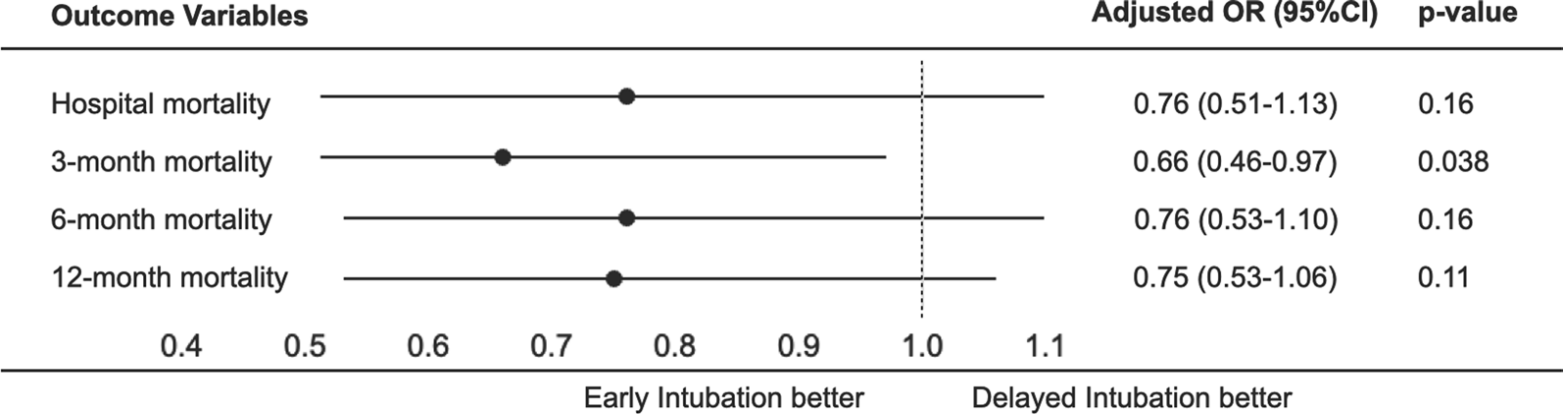
Comparison of mortality outcomes—including in-hospital, 3-month, 6-month, and 12-month mortality—between early intubation and delayed intubation with multivariable logistic regression analysis. *OR* adjusted odds ratios, *CI* confidence intervals

A total of 233 people (18.9%) died in hospital. In-hospital mortality was similar between the two groups (19.2% vs. 17.5%; p = 0.52) (Table 2). After adjusting for baseline covariates identified by DAGs (Supplementary Fig. 1), in-hospital mortality did not differ (adjusted OR = 0.76; 95% CI 0.51–1.13; p = 0.16) between early intubation group and delayed intubation group (Table 3). Figure 2 illustrates the primary and secondary mortality outcome (in-hospital, 3-, 6-, and 12-month) comparisons between early and delayed intubation groups using adjusted multivariable logistic regression, and presented as forest plots.

**Table 2:** Unadjusted primary and secondary outcomes

	Early intubation (n = 983)	Delayed intubation (n = 252)	Total (n = 1235)	P-value
Primary outcome				
In-hospital mortality	189 (19.2)	44 (17.5)	233 (18.9)	0.52
Secondary outcomes				
ICU mortality	131 (13.3)	32 (12.7)	163 (13.2)	0.32
Duration of IMV (hours)	43.0 (16.0–104.0)	38.5 (13.0–95.5)	42.0 (16.0–100.0)	0.52
Tracheostomy	34 (3.5)	5 (2.0)	39 (3.2)	< 0.001
Length of stay (days)				
ICU length of stay	4.5 (2.5–8.0)	6.6 (3.7–11.1)	4.8 (2.7–8.6)	< 0.001
Hospital length of stay	12.1 (6.1–21.9)	16.6 (10.4–29.2)	13.0 (6.9–23.5)	0.027
Post-ICU length of stay	6.3 (1.9–14.0)	8.6 (3.2–18.1)	6.8 (2.1–15.1)	0.57
Hospital outcomes (excluding mortality)				
Discharged home	487 (49.5)	127 (50.4)	614 (49.7)	0.88
Transferred to other hospital	170 (17.3)	40 (15.9)	210 (17.0)	
Rehabilitation facility	78 (7.9)	25 (9.9)	103 (8.3)	
Chronic care facility/Nursing home	14 (5.6)	52 (5.3)	66 (5.3)	
Home	2 (0.8)	7 (0.7)	9 (0.7)	
ICU complications				
Delirium	121 (12.3)	37 (14.7)	158 (12.8)	0.40
Pressure Injury	17 (6.7)	50 (5.1)	67 (5.4)	0.49
Long-term survival probabilities, % (95% CI)				
3-month	80.3 (77.9–82.9)	79.2 (74.3–84.4)		
6-month	77.0 (74.4–79.7)	77.1 (74.3–82.5)		
12-month	74.8 (72.1–77.6)	73.8 (68.5–79.6)		

Data are n (%), mean (SD), or median [IQR]

*ICU* intensive care unit, *IMV* invasive mechanical ventilation, *IQR* inter-quartile range, *SD* standard deviation, *CI* confidence intervals

Statistically significant differences between groups (P < 0.05) were observed for ICU length of stay, hospital length of stay, and receipt of tracheostomy

**Table 3:** Comparison of primary outcomes (in-hospital mortality) between early intubation and delayed intubation with multivariable logistic regression analysis

	OR (95% CI)	P-value
Delayed intubation	Reference group	
Early intubation	0.76 (0.51–1.13)	0.16
Age	1.02 (1.01–1.03)	0.001
SOFA score	1.23 (1.17–1.29)	< 0.001
Non-frail (CFS 1–4)	Reference group	
Frailty (CFS 5–8)	1.79 (1.23–2.61)	0.004
Unknown frailty	1.65 (1.12–2.44)	0.012
Hospital admission source		
Other hospital (without ED)	0.82 (0.48–1.40)	0.48
Other healthcare facilities	0.34 (0.11–1.04)	0.05
Rehabilitation	NA	0.99
Other hospital-ED	0.86 (0.53–1.39)	0.53
Treatment limitations at ICU admission	2.42 (1.59–3.70)	< 0.001
Emergency response admission	1.37 (1.03–1.82)	0.031

*OR* adjusted odds ratios, *CI* confidence intervals, *SOFA* Sequential Organ Failure Assessment, *CFS* clinical frailty scale, *ED* emergency department, *ICU* intensive care unit

The findings were consistent with the main analysis in the two sensitivity analyses using the APACHE III score, both as is and individual components, excluding GCS and respiratory-related variables (adjusted OR = 0.79; 95% CI 0.53–1.18 and 0.96; 95% CI 0.64–1.43, respectively, see Supplementary Tables 2 and 3). Furthermore, in a sensitivity analysis excluding patients discharged within the first 24 h of ICU admission (n = 82), the association between intubation timing and in-hospital mortality remained statistically non-significant after multivariable adjustment (OR: 0.70, 95% CI 0.46–1.05). These findings were consistent with the primary analysis.

### Subgroup analysis

When comparing with patients aged < 65 years (adjusted OR = 0.73; 95% CI 0.36–1.47), there were no significant differences in the association of early intubation with in-hospital mortality in patients aged ≥ 65 years (adjusted OR = 0.76; 95% CI 0.46–1.24; Supplementary Table 4). Early intubation was associated with lower in-hospital mortality in the subgroup of patients who were transferred to the ICU from a general ward (adjusted OR = 0.50; 95% CI 0.28–0.89; Supplementary Table 5). However, there were no significant differences in the association of early intubation with in-hospital mortality based on ICU admission source (interaction p = 0.06; Supplementary Fig. 2). Early intubation was not associated with lower in-hospital mortality in other subgroup analyses, including sex, vasopressor use, and frailty (Supplementary Tables 6–8, Supplementary Fig. 2).

### Secondary outcomes

Table 2 shows the raw secondary outcomes between the early intubation group and the delayed intubation group. The early intubation group had shorter hospital length of stays (12.1 [6.1–21.9] vs. 16.6 [10.4–29.2] days, p = 0.027), and ICU length of stays (4.5 [2.5–8.0] vs. 6.6 [3.7–11.1] days, p < 0.001), when compared with the delayed intubation group. Although the duration of MV was comparable between the two groups (43.0 [16.0, 104.0] vs. 38.5 [13.0, 95.5] hours, p = 0.52), early intubation group more frequently required tracheostomy (3.5% vs. 2.0%; p < 0.001) than delayed intubation group. There were no differences in the frequency of delirium, pressure injury or non-home discharge destination between the two groups.

**Fig. 3:**
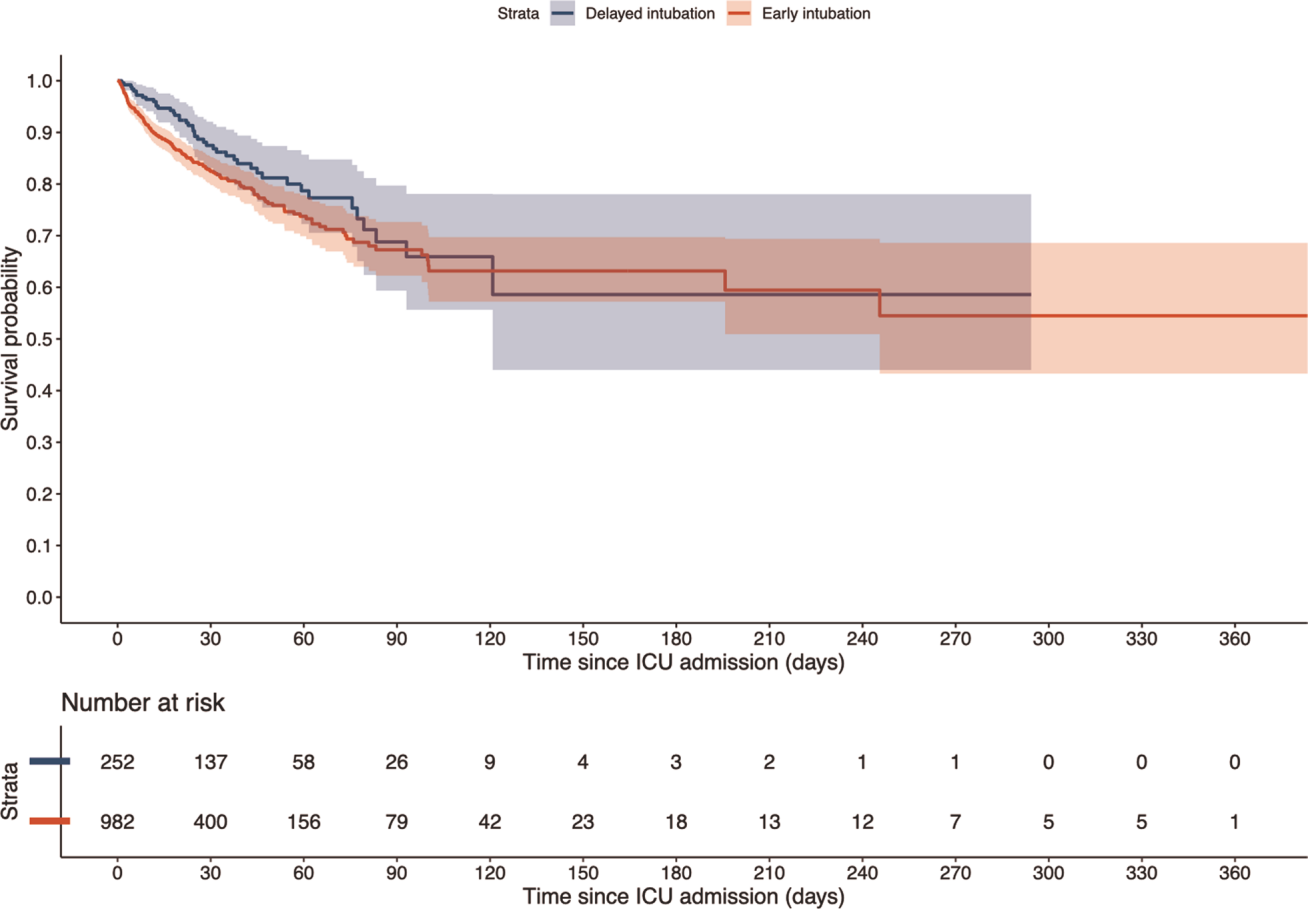
Unadjusted survival curves of early intubation patients and delayed intubation patients from initial admission to ICU until follow-up of up to 1 year. *ICU* intensive care unit

Figure 3 presents unadjusted Kaplan Meier survival curves comparing early and delayed intubation groups from ICU admission up to 1-year follow-up. While delayed intubation was associated with better survival at 3 months, long-term survival was comparable between the two groups. In multivariable logistic regression analysis adjusting for the same confounders as the main analysis, the early intubation group was associated with lower mortality only at 3 months of mortality when compared with the delayed intubation group (adjusted OR = 0.66; 95% CI 0.46–0.97; Fig. 2, Supplementary Table 9). However, there were no significant differences in the mortality at 6 months (adjusted OR = 0.76; 95% CI 0.53–1.10) and 12 months (adjusted OR = 0.75; 95% CI 0.53–1.06) between the two groups. In adjusted linear regression, early intubation was associated with lower ICU length of stay (adjusted point estimate = −2.94 days; 95% CI −3.90 to −1.98; Supplementary Table 10). However, early intubation was not associated with lower hospital length of stay in the adjusted analysis (adjusted point estimate = −3.05 days; 95% CI −6.89 to 0.80; Supplementary Table 11).

## Discussion

This multicenter retrospective cohort study found that early intubation (within 24 h from ICU admission) was not associated with lower in-hospital mortality. This finding was also consistent across the sensitivity analysis and various pre-defined subgroup analyses: based on age, sex, frailty status, presence of shock, and the source of ICU admission. Early intubation was associated with lower in-hospital mortality only in the subgroup of patients who were transferred to the ICU from a general ward; however, given the retrospective design, the potential for unmeasured confounding, and the non-significant interaction test between admission source and intubation timing (p = 0.06), these findings should be considered hypothesis-generating and require confirmation in prospective studies. Although early intubation was associated with adjusted lower ICU length of stay, when compared to patients with delayed intubation, there was no adjusted association for hospital length of stay. While the 3-month mortality was higher in patients with early intubation, there was no difference at 6- and 12-months between the two groups.

While evidence suggests potential benefits of early initiation of MV in critical care [17], most have focused on patients with acute respiratory failure [2, 18], including COVID-19 [19, 20]. Contrarily, only a few studies have examined cohorts of patients with sepsis, and their findings have been inconsistent [4–7], potentially due to the heterogeneity of sepsis as a syndrome in and of itself, and its various presentations in each organ system [21]. Previous studies [4–7] included patients with various etiologies of sepsis, potentially contributing to variability in outcomes. In this study, we sought to minimize variability in treatment effects due to heterogeneity by restricting the study population. To our knowledge, this is the first study to focus specifically on urosepsis and evaluate the impact of early intubation.

Early intubation was not associated with improved in-hospital, 6-month, or 12-month mortality, and notably, 3-month mortality was higher compared to delayed intubation. Our findings indicate that intubation timing may not be pivotal in urosepsis care. In addition, a higher tracheostomy rate was observed in the early intubation group. This may have reflected greater baseline illness severity and organ dysfunction influencing clinical decision-making, rather than differences in ventilator duration, and should therefore be interpreted with caution. On the other hand, the observed reduction in ICU length of stay with early intubation, although an exploratory secondary outcome, is of interest and appears consistent with previous literature [7, 9]. However, this association should be interpreted cautiously, as it may reflect differences in illness trajectory rather than a direct effect of intubation timing. Previous studies have reported that delayed intubation can lead to respiratory muscle fatigue, aspiration, and progressive hypoxemia, all of which may prolong ICU admission [22, 23]. Thus, while our findings are hypothesis-generating and compatible with the hypothesis that prompt cardiorespiratory stabilization could contribute to shorter ICU stays, causality cannot be inferred from our retrospective analysis. Ultimately, we were unable to demonstrate the expected benefits of early intubation to the extent that has been anticipated in patients with respiratory failure.

Our findings have a few important clinical implications. While early intubation did not influence in-hospital or long-term mortality, it was associated with shorter ICU length of stay, which may suggest possible benefits in ICU resource utilization and patient flow. Furthermore, the lack of benefits across predefined subgroups, including age, frailty, and shock status, suggests possible consistency of the findings across diverse ICU populations with urosepsis. This may support a nuanced, patient-specific approach rather than a blanket early or delayed strategy. Future prospective randomized controlled trials are needed to understand the optimal timing of intubation to maximize patient outcomes with urosepsis.

There are several strengths to this study. First, our study included patients admitted to most ICUs across Australia over 5 years and is thus representative of practices and outcomes. The linkage success to the death register was high, facilitating an accurate assessment of long-term survival. Second, by restricting the study population, we minimized variability in treatment effects due to heterogeneity. Third, the results of the primary analyses were consistent across both the sensitivity and subgroup analyses, lending further support to the robustness of our findings. Finally, we observed not only short-term outcomes but also medium- to long-term outcomes.

However, our study has several potential limitations. First, our analysis is based on retrospective registry data, and as such, we cannot estimate causal effects. The ANZICS-APD registry is limited by missing data and potential inaccuracies in data collection or coding despite a robust training and credentialing scheme as part of the ANZICS-APD. In the absence of site-based auditing, the extent of any potential misclassification remains uncertain. Although we believe that our cohort is broadly representative of the larger population, this cannot be confirmed. Furthermore, key clinical factors—such as clinical indications for intubation, causative pathogens and resistance profiles, the underlying cause of urinary tract infection (e.g., stones), and interventions like ureteral stenting or nephrostomy—were not available, potentially contributing to residual confounding. Second, our analysis was limited to patients who underwent intubation, excluding those who were not intubated. While this approach allowed us to focus on critically ill patients for whom the timing of intubation was a genuine clinical concern, it may limit the generalizability of our findings to all patients with urosepsis. Third, due to the insufficient information in our intubation data, adjustment for immortal time bias was not possible. Patients who would have been classified into the delayed intubation group in a prospective study may have died prior to that point and, therefore, were not included in the analysis. In such cases, delayed intubation may have been overestimated. To address potential immortal time bias, we conducted a sensitivity analysis excluding patients discharged within 24 h of ICU admission. Results were consistent with the primary analysis, indicating that the observed associations were unlikely to be materially influenced by early deaths and supporting the robustness of our findings. Fourth, our comparison of ICU and hospital lengths of stay between early and delayed intubation groups may be subject to structural bias. By definition, patients in delayed group were not intubated until after 24 h, which may inherently extend their total length of stay irrespective of clinical course. Although we adjusted for illness severity and baseline characteristics in our multivariable models, residual confounding and structural bias cannot be entirely excluded. Nevertheless, the duration of mechanical ventilation did not significantly differ between groups, suggesting that the post-intubation clinical course was broadly comparable. Furthermore, the definition of early intubation varies between studies, which we consider a challenge in this field [17]. If more granular data—such as hourly timing—were available, it might allow for a more precise classification and more robust conclusions. Finally, the possibility of confounding by indication should be considered. Patient severity at admission not only affects outcomes but also influences the decision to intubate early. Although we adjusted for severity using composite scores such as SOFA and APACHE, key variables, including oxygenation parameters, GCS, and lactate, were not individually incorporated in the regression models, which may limit causal inference. The possibility of residual and uncontrolled confounding cannot be excluded.

## Conclusion

In this multicenter cohort study, early intubation and MV within 24 h of ICU admission in patients with urosepsis were not associated with improved in-hospital or long-term mortality. These findings provide important insights for future research regarding the optimal timing of intubation in urosepsis.

## Supplementary Information

Supplementary material 1: Figure 1. Causal-directed acyclic graph. GCS, Glasgow coma scale.

Supplementary material 2: Figure 2. Forest plot presenting adjusted odds ratios with 95% confidence intervals for in-hospital mortality, comparing early versus delayed intubation across subgroups using multivariable logistic regression analyses. Interaction p-values are shown to assess heterogeneity of treatment effect across subgroups. OR, adjusted odds ratios; CI, confidence intervals; CFS, clinical frailty scale; ED, emergency department.

Supplementary material 3.

## Data Availability

The data that support the findings of this study are available from the corresponding author upon reasonable request.

## Abbreviations

MV: Mechanical ventilation
ICU: Intensive care unit
ANZICS: Australian and New Zealand Intensive Care Society
APACHE: Acute Physiological and Chronic Health Evaluation
ANZICS-APD: ANZICS-adult patient database
CFS: Clinical frailty scale
SOFA: Sequential organ failure assessment
ANZROD: Australia New Zealand Risk of Death mortality prediction model)
ECMO: Extracorporeal membrane oxygenation
SD: Standard deviation
IQR: Interquartile range
OR: Odds ratio
95% CI: 95% Confidence interval
DAG: Directed acyclic graph
ED: Emergency department
GCS: Glasgow Coma Scale

## References

[1] Kranz J, Bartoletti R, Bruyere F, Cai T, Geerlings S, Koves B, et al. European association of urology guidelines on urological infections: summary of the 2024 guidelines. Eur Urol. 2024;86(1):27–41. 38714379 10.1016/j.eururo.2024.03.035

[2] Tandogdu Z, Koves B, Ristovski S, Balci MBC, Rennesund K, Gravas S, et al. Urosepsis 30-day mortality, morbidity, and their risk factors: SERPENS study, a prospective, observational multi-center study. World J Urol. 2024;42(1):314. 38730089 10.1007/s00345-024-04979-2 PMC11087335

[3] Bauer PR, Kumbamu A, Wilson ME, Pannu JK, Egginton JS, Kashyap R, et al. Timing of intubation in acute respiratory failure associated with sepsis: a mixed methods study. Mayo Clin Proc. 2017;92(10):1502–10. 28867256 10.1016/j.mayocp.2017.07.001

[4] Delbove A, Darreau C, Hamel JF, Asfar P, Lerolle N. Impact of endotracheal intubation on septic shock outcome: a post hoc analysis of the SEPSISPAM trial. J Crit Care. 2015;30(6):1174–8. 26410680 10.1016/j.jcrc.2015.08.018

[5] Yang T, Shen Y, Park JG, Schulte PJ, Hanson AC, Herasevich V, et al. Outcome after intubation for septic shock with respiratory distress and hemodynamic compromise: an observational study. BMC Anesthesiol. 2021;21(1):253. 34696738 10.1186/s12871-021-01471-x PMC8543776

[6] Mellado-Artigas R, Ferrando C, Martino F, Delbove A, Ferreyro BL, Darreau C, et al. Early intubation and patient-centered outcomes in septic shock: a secondary analysis of a prospective multicenter study. Crit Care. 2022;26(1):163. 35672860 10.1186/s13054-022-04029-6 PMC9171484

[7] Kim G, Oh DK, Lee SY, Park MH, Lim CM, Korean Sepsis Alliance i. Impact of the timing of invasive mechanical ventilation in patients with sepsis: a multicenter cohort study. Crit Care. 2024;28(1):297. 39252133 10.1186/s13054-024-05064-1 PMC11385489

[8] Asfar P, Meziani F, Hamel JF, Grelon F, Megarbane B, Anguel N, et al. High versus low blood-pressure target in patients with septic shock. N Engl J Med. 2014;370(17):1583–93. 24635770 10.1056/NEJMoa1312173

[9] Darreau C, Martino F, Saint-Martin M, Jacquier S, Hamel JF, Nay MA, et al. Use, timing and factors associated with tracheal intubation in septic shock: a prospective multicentric observational study. Ann Intensive Care. 2020;10(1):62. 32449053 10.1186/s13613-020-00668-6 PMC7245631

[10] Silva PL, Ball L, Rocco PRM, Pelosi P. Physiological and pathophysiological consequences of mechanical ventilation. Semin Respir Crit Care Med. 2022;43(3):321–34. 35439832 10.1055/s-0042-1744447

[11] Kobayashi H, Uchino S, Takinami M, Uezono S. The impact of ventilator-associated events in critically ill subjects with prolonged mechanical ventilation. Respir Care. 2017;62(11):1379–86. 28720671 10.4187/respcare.05073

[12] Curley GF, Laffey JG, Zhang H, Slutsky AS. Biotrauma and ventilator-induced lung injury: clinical implications. Chest. 2016;150(5):1109–17. 27477213 10.1016/j.chest.2016.07.019

[13] Shehabi Y, Bellomo R, Reade MC, Bailey M, Bass F, Howe B, et al. Early intensive care sedation predicts long-term mortality in ventilated critically ill patients. Am J Respir Crit Care Med. 2012;186(8):724–31. 22859526 10.1164/rccm.201203-0522OC

[14] Evans L, Rhodes A, Alhazzani W, Antonelli M, Coopersmith CM, French C, et al. Surviving sepsis campaign: International Guidelines for Management of Sepsis and Septic Shock 2021. Crit Care Med. 2021;49(11):e1063–143. 34605781 10.1097/CCM.0000000000005337

[15] Evaluation ACfOaR. Adult Patient Database Data Dictionary: Australian and New Zealand Intensive Care Society; 2022 [Available from: https://www.anzics.com.au/wp-content/uploads/2021/03/ANZICS-APD-Dictionary-Version-6.1.pdf. Accessed 1 Jan 2025]

[16] Singer M, Deutschman CS, Seymour CW, Shankar-Hari M, Annane D, Bauer M, et al. The third international consensus definitions for sepsis and septic shock (Sepsis-3). JAMA. 2016;315(8):801–10. 26903338 10.1001/jama.2016.0287 PMC4968574

[17] Xixi NA, Kremmydas P, Xourgia E, Giannopoulou V, Sarri K, Siempos II. Association between timing of intubation and clinical outcomes of critically ill patients: a meta-analysis. J Crit Care. 2022;71:154062. 35588639 10.1016/j.jcrc.2022.154062

[18] Dumas G, Lemiale V, Rathi N, Cortegiani A, Pene F, Bonny V, et al. Survival in immunocompromised patients ultimately requiring invasive mechanical ventilation: a pooled individual patient data analysis. Am J Respir Crit Care Med. 2021;204(2):187–96. 33751920 10.1164/rccm.202009-3575OC

[19] Mellado-Artigas R, Ferreyro BL, Angriman F, Hernandez-Sanz M, Arruti E, Torres A, et al. High-flow nasal oxygen in patients with COVID-19-associated acute respiratory failure. Crit Care. 2021;25(1):58. 33573680 10.1186/s13054-021-03469-w PMC7876530

[20] Papoutsi E, Giannakoulis VG, Xourgia E, Routsi C, Kotanidou A, Siempos II. Effect of timing of intubation on clinical outcomes of critically ill patients with COVID-19: a systematic review and meta-analysis of non-randomized cohort studies. Crit Care. 2021;25(1):121. 33766109 10.1186/s13054-021-03540-6 PMC7993905

[21] Fohner AE, Greene JD, Lawson BL, Chen JH, Kipnis P, Escobar GJ, et al. Assessing clinical heterogeneity in sepsis through treatment patterns and machine learning. J Am Med Inform Assoc. 2019;26(12):1466–77. 31314892 10.1093/jamia/ocz106 PMC7647146

[22] Kangelaris KN, Ware LB, Wang CY, Janz DR, Zhuo H, Matthay MA, et al. Timing of intubation and clinical outcomes in adults with acute respiratory distress syndrome. Crit Care Med. 2016;44(1):120–9. 26474112 10.1097/CCM.0000000000001359 PMC4774861

[23] Mosier JM, Sakles JC, Whitmore SP, Hypes CD, Hallett DK, Hawbaker KE, et al. Failed noninvasive positive-pressure ventilation is associated with an increased risk of intubation-related complications. Ann Intensive Care. 2015;5:4. 25852964 10.1186/s13613-015-0044-1 PMC4385202

