# Supplementary figures and images for: Effect of early intubation on patient-centered outcomes in urosepsis: a retrospective multicenter cohort study
Source: J Intensive Care. 2025 Oct 23;13:58. doi: 10.1186/s40560-025-00829-4 (PMC12548251; doi:10.1186/s40560-025-00829-4)

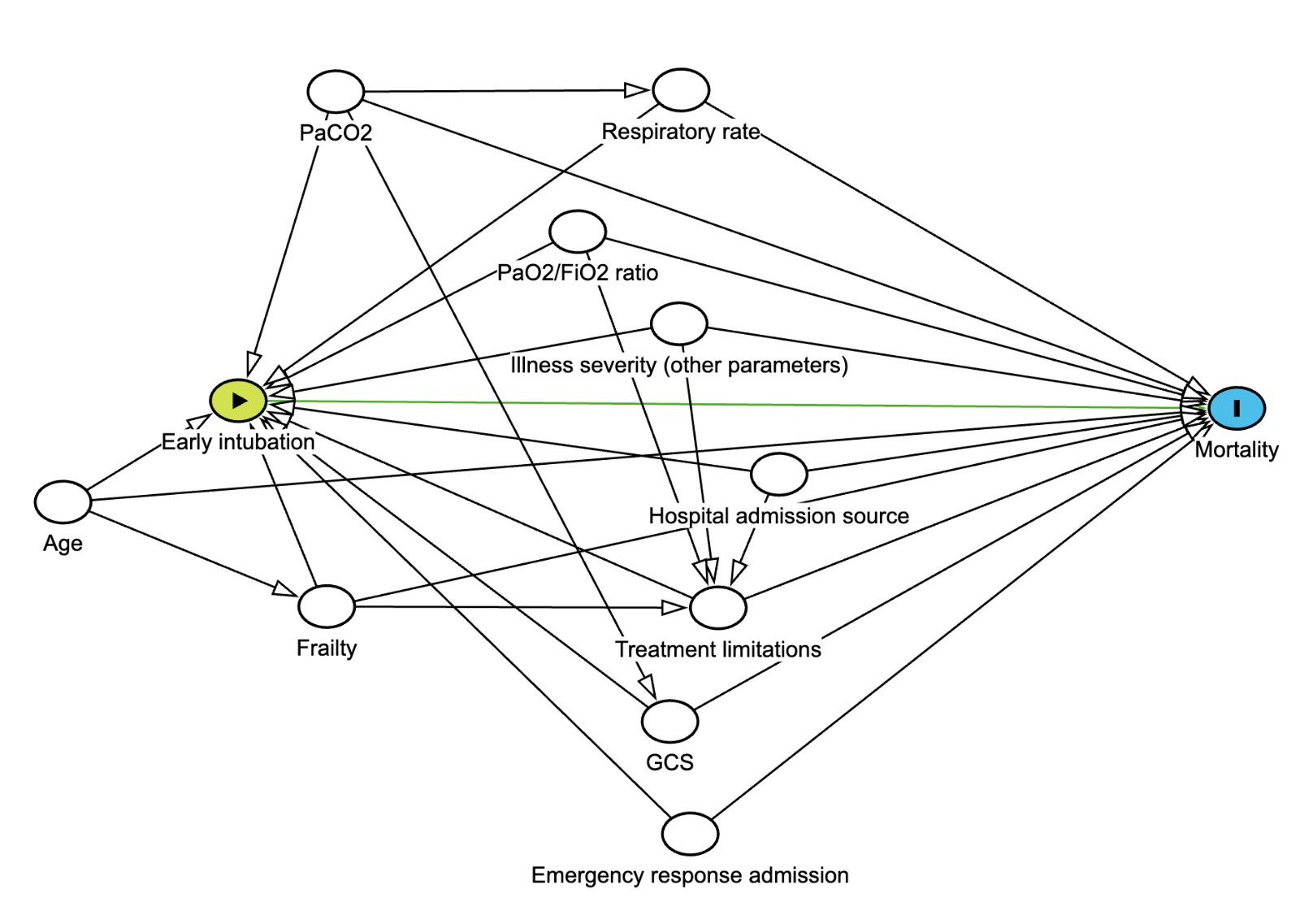

Supplement: Supplementary file 1 — Supplementary material 1: Figure 1. Causal-directed acyclic graph. GCS, Glasgow coma scale. [file 40560_2025_829_MOESM1_ESM.tiff]

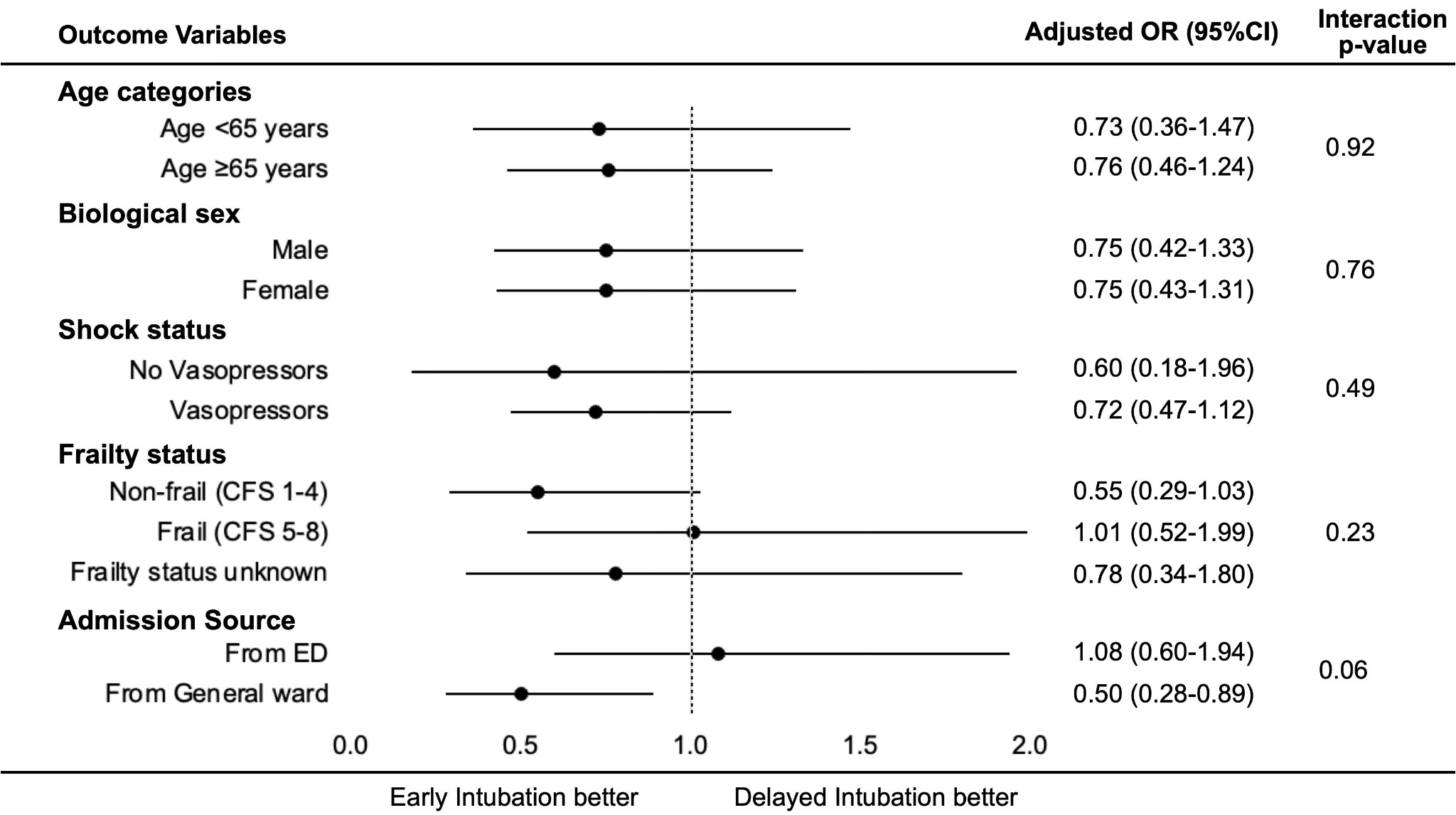

Supplement: Supplementary file 2 — Supplementary material 2: Figure 2. Forest plot presenting adjusted odds ratios with 95% confidence intervals for in-hospital mortality, comparing early versus delayed intubation across subgroups using multivariable logistic regression analyses. Interaction p-values are shown to assess heterogeneity of treatment effect across subgroups. OR, adjusted odds ratios; CI, confidence intervals; CFS, clinical frailty scale; ED, emergency department. [file 40560_2025_829_MOESM2_ESM.tiff]
