# Supplementary material for: Effect of early intubation on patient-centered outcomes in urosepsis: a retrospective multicenter cohort study
Source: J Intensive Care. 2025 Oct 23;13:58. doi: 10.1186/s40560-025-00829-4 (PMC12548251; doi:10.1186/s40560-025-00829-4)
Supplement: Supplementary file 3 — Supplementary material 3. [file 40560_2025_829_MOESM3_ESM.docx]

**Supplementary Table 1.**

**Patient characteristics of all urosepsis patients (not intubated and intubated)**

| **Demographic variable** | **Not intubated**  **(n = 13,459)** | **Intubated**  **(n = 1,235)** | **P-value** |
| --- | --- | --- | --- |
| Age (years) | 71.1 [59.0-79.6] | 67.8 [55.8-76.0] | <0.001 |
| Female sex | 6,986 (51.9) | 659 (53.4) | 0.339 |
| BMI (kg m^-2^)* | 27.5 [23.7-32.9] | 29.4 [25.3-35.6] | 0.449 |
| Indigenous status^†^ | 879 (6.5) | 73 (5.9) | 0.619 |
| Hospital classification  Rural/Regional  Metropolitan  Tertiary/Teaching  Private | 3,103 (23.1)  3,874 (28.8)  4,733 (35.2)  1,749 (13.0) | 175 (14.2)  318 (25.7)  650 (52.6)  92 (7.4) | <0.001 |
| Hospital admission source  Home/Hospital in the home  Other hospital  Other healthcare facilities^‡^ | 10,625 (78.9)  2,296 (17.1)  538 (4.0) | 897 (72.6)  296 (24.0)  42 (3.4) | <0.001 |
| Comorbidities  Chronic respiratory condition  Chronic cardiovascular condition  Chronic renal failure  Chronic liver disease  Diabetes mellitus  Immunosuppressive therapy  Metastatic cancer  Lymphoma  Leukemia | 961 (7.1)  1,478 (11.0)  745 (5.5)  217 (1.6)  4,063 (30.2)  1,294 (9.6)  855 (6.4)  203 (1.5)  206 (1.5) | 100 (8.1)  116 (9.4)  72 (5.8)  36 (2.9)  439 (35.5)  90 (7.3)  43 (3.5)  15 (1.2)  13 (1.1) | 0.384  0.163  0.776  0.005  <0.001  0.007  <0.001  0.727  0.389 |
| APACHE II score | 18.0 [13.0-22.0] | 25.0 [20.0-31.0] | <0.001 |
| APACHE III score | 60.0 [46.0-74.0] | 81.0 [64.0-102.0] | <0.001 |
| ANZROD score (%) | 7.0 (10.8) | 21.0 (22.1) | <0.001 |
| SOFA score | 4.0 [3.0, 6.0] | 7.0 [5.0, 10.0] | <0.001 |
| CFS categories  CFS 1-4, non-frail  CFS 5-8, frail | 6,576 (48.9)  4,279 (31.8) | 547 (44.3)  362 (29.3) | <0.001 |
| Treatment limitation at ICU admission | 2,956 (22.0) | 146 (11.8) | <0.001 |
| Organ supports during ICU stay |  |  |  |
| Non-invasive ventilation  Vasopressors  ECMO  Renal replacement therapy | 935 (6.9)  8,472 (62.9)  0 (0.0)  508 (3.8) | 253 (20.5)  984 (79.7)  3 (0.2)  311 (25.2) | <0.001  <0.001  <0.001  <0.001 |
| Total bilirubin (mg/dL) | 12.0 [7.0-19.0] | 16.0 [9.0-28.0] | <0.001 |
| Platelets (10^3^/μL) | 163.0 [116.0-223.0] | 151.0(85.0, 220.0) | <0.001 |
| Potassium (mEq/L) | 4.2 [3.9-4.7] | 4.5 [4.2-5.1] | <0.001 |
| Creatinine (μmol/L) | 126.0 [76.0-202.0] | 190.0 [116.0-293.0] | <0.001 |
| Vital signs (worstest within 24 hours) |  |  |  |
| P/F ratio | 319.0 [245.7-376.2] | 215.0 [144.7-310.0] | <0.001 |
| GCS | 15.0 [14.0-15.0] | 14.0 [11.0-15.0] | <0.001 |
| MAP (mmHg) | 62.0 [56.0-68.0] | 61.0 [55.0-66.0] | 0.734 |
| 24 hours urine output (ml) | 1,832.0 [1,163.0- 2,745.0] | 1,354.0 [596.0-2,220.0] | <0.001 |

Data are n (%), mean [SD], or median (IQR).

BMI, body mass index; ICU, intensive care unit; ED, emergency department; APACHE, Acute Physiology and Chronic Health Evaluation; ANZROD, Australia New Zealand Risk of death; SOFA, Sequential Organ Failure Assessment; CFS, clinical frailty scale; ECMO, extracorporeal membrane oxygenation; P/F ratio, PaO2/FiO2 ratio; GCS, Glasgow coma scale; MAP, mean arterial pressure; IQR, inter-quartile range; SD, standard deviation.

* BMI: We had data only available on height or weight

^†^Indigenous: the patient identifies as indigenous to the country where they receive treatment. In Australia, a patient who identifies as Aboriginal or Torres Strait Islander should be coded as indigenous.

^‡^Other healthcare facilities included Nursing home, Chronic care, Palliative care, Rehabilitation

**Supplementary Table 2.**

**Sensitivity analysis: Multivariable logistic regression of in-hospital mortality comparing early vs delayed intubation, adjusted for APACHE III score, Clinical Frailty Scale categories, and emergency response admission**

|  |  |  |
| --- | --- | --- |
|  | **OR (95% CI)** |  |
| Delayed intubation | Reference group |  |
| Early intubation | 0.79 (0.53–1.18) |  |
| APACHE III score | 1.03 (1.03–1.04) |  |
| Non-frail (CFS 1-4) | Reference group |  |
| Frailty (CFS 5-8) | 1.76 (1.21–2.55) |  |
| Unknown frailty | 1.61 (1.08–2.40) |  |
| Emergency response admission | 1.52 (1.14–2.02) |  |

OR, adjusted odds ratios; CI, confidence intervals; CFS, clinical frailty scale; ED, emergency department; ICU, intensive care unit.

**Supplementary Table 3.**

**Sensitivity analysis: Multivariable logistic regression of in-hospital mortality comparing early vs delayed intubation, adjusted for APACHE III score, excluding the Glasgow Coma Scale and respiratory-related components (respiratory rate and oxygenation variables), Clinical Frailty Scale categories, and emergency response admission**

|  | **OR (95% CI)** |
| --- | --- |
| Delayed intubation | Reference group |
| Early intubation | 0.96 (0.64–1.43) |
| *APACHE III score | 1.04 (1.04–1.05) |
| Non-frail (CFS 1-4) | Reference group |
| Frailty (CFS 5-8) | 1.89 (1.30–2.74) |
| Unknown frailty | 1.55 (1.04–2.32) |
| Emergency response admission | 1.56 (1.17–2.07) |

OR, adjusted odds ratios; CI, confidence intervals; CFS, clinical frailty scale; ED, emergency department; ICU, intensive care unit.

* Individual components of the APACHE III score, excluding the GCS and respiratory-related components (respiratory rate and oxygenation variables)

**Supplementary Table 4.**

**Subgroup analysis by age categories comparing primary outcomes (in-hospital mortality) between early intubation and delayed intubation**

|  | **Age < 65 years** | | **Age ≥ 65 years** | |
| --- | --- | --- | --- | --- |
|  | **OR (95% CI)** | **P-value** | **OR (95% CI)** | **P-value** |
| Delayed intubation | Reference group |  | Reference group |  |
| Early intubation | 0.73 (0.36-1.47) | 0.32 | 0.76 (0.46-1.24) | 0.29 |
| Age | 1.01 (0.99-1.04) | 0.34 | 1.01 (0.98-1.04) | 0.65 |
| SOFA score | 1.26 (1.15-1.37) | <0.0001 | 1.22 (1.15-1.30) | <0.0001 |
| Non-frail (CFS 1-4) | Reference group |  | Reference group |  |
| Frailty (CFS 5-8) | 2.39 (1.17-4.86) | 0.038 | 1.58 (1.01-2.46) | 0.044 |
| Unknown frailty | 2.17 (1.05-4.48) | 0.031 | 1.47 (0.93-2.33) | 0.098 |
| Hospital admission source |  |  |  |  |
| other hospital (without ED) | 0.54 (0.18-1.69) | 0.36 | 0.91 (0.50-1.68) | 0.75 |
| other healthcare facilities | 0.78 (0.14-4.19) | 0.75 | 0.22 (0.05-1.01) | 0.051 |
| rehabilitation | NA | 1.00 | NA | 0.99 |
| other hospital-ED | 0.86 (0.34-2.14) | 0.82 | 0.83 (0.47-1.47) | 0.48 |
| Treatment limitations at ICU admission | 2.30 (0.83-6.38) | 0.078 | 2.44 (1.53-3.89) | 0.0002 |
| Emergency response admission | 1.16 (0.69-1.97) | 0.56 | 1.45 (1.04-2.02) | 0.034 |

OR, adjusted odds ratios; CI, confidence intervals; SOFA, Sequential Organ Failure Assessment; CFS, clinical frailty scale; ED, emergency department; ICU, intensive care unit; GCS, Glasgow coma scale.

**Supplementary Table 5.**

**Subgroup analysis by admission source comparing primary outcomes (in-hospital mortality) between early intubation and delayed intubation**

|  | **via ED** | | **via general ward** | |
| --- | --- | --- | --- | --- |
|  | **OR (95% CI)** | **P-value** | **OR (95% CI)** | **P-value** |
| Delayed intubation | Reference group |  | Reference group |  |
| Early intubation | 1.08 (0.60–1.94) | 0.82 | 0.50 (0.28–0.89) | 0.018 |
| Age | 1.02 (1.01–1.04) | 0.0079 | 1.02 (1.00-1.04) | 0.025 |
| SOFA score | 1.22 (1.14–1.30) | <0.0001 | 1.25 (1.15-1.36) | <0.0001 |
| Non-frail (CFS 1-4) | Reference group |  | Reference group |  |
| Frailty (CFS 5-8) | 1.58 (0.96–2.61) | 0.076 | 2.33 (1.29–4.22) | 0.010 |
| Unknown frailty | 1.32 (0.77–2.25) | 0.29 | 2.09 (1.17–3.73) | 0.017 |
| Hospital admission source |  |  |  |  |
| other hospital (without ED) | 1.66 (0.60–4.63) | 0.35 | 0.41 (0.20–0.82) | 0.010 |
| other healthcare facilities | NA | 0.99 | 0.62 (0.17–2.22) | 0.45 |
| rehabilitation | NA | 1.00 | NA | 0.99 |
| other hospital-ED | 1.34 (0.66–2.69) | 0.40 | 0.42 (0.20–0.87) | 0.017 |
| Treatment limitations at ICU admission | 2.05 (1.11–3.78) | 0.021 | 2.84 (1.55–5.22) | 0.0008 |
| Emergency response admission | 1.15 (0.72–1.86) | 0.53 | 0.87 (0.54–1.41) | 0.45 |

OR, adjusted odds ratios; CI, confidence intervals; SOFA, Sequential Organ Failure Assessment; CFS, clinical frailty scale; ED, emergency department; ICU, intensive care unit; GCS, Glasgow coma scale.

**Supplementary Table 6.**

**Subgroup analysis by sex comparing primary outcomes (in-hospital mortality) between early intubation and delayed intubation**

|  | **Male** | | **Female** | |
| --- | --- | --- | --- | --- |
|  | **OR (95% CI)** | **P-value** | **OR (95% CI)** | **P-value** |
| Delayed intubation | Reference group |  | Reference group |  |
| Early intubation | 0.75 (0.42-1.33) | 0.27 | 0.75 (0.43-1.31) | 0.29 |
| Age | 1.03 (1.01-1.05) | 0.0027 | 1.02 (1.00-1.03) | 0.066 |
| SOFA score | 1.23 (1.15-1.32) | <0.0001 | 1.24 (1.16-1.34) | <0.0001 |
| Non-frail (CFS 1-4) | Reference group |  | Reference group |  |
| Frailty (CFS 5-8) | 2.11 (1.22-3.66) | 0.0098 | 1.45 (0.85-2.46) | 0.22 |
| Unknown frailty | 1.69 (0.97-2.93) | 0.063 | 1.62 (0.92-2.83) | 0.092 |
| Hospital admission source |  |  |  |  |
| other hospital (without ED) | 0.85 (0.36-2.01) | 0.68 | 0.81 (0.40-1.62) | 0.60 |
| other healthcare facilities | 0.57 (0.15-2.16) | 0.43 | 0.14 (0.02-1.22) | 0.069 |
| rehabilitation | NA | 0.99 | NA | 0.99 |
| other hospital-ED | 0.60 (0.28-1.25) | 0.17 | 1.22 (0.64-2.33) | 0.60 |
| Treatment limitations at ICU admission | 1.48 (0.80-2.74) | 0.20 | 4.33 (2.35-7.98) | <0.0001 |
| Emergency response admission | 1.62 (1.07-2.46) | 0.024 | 1.22 (0.64-2.33) | 0.41 |

OR, adjusted odds ratios; CI, confidence intervals; SOFA, Sequential Organ Failure Assessment; CFS, clinical frailty scale; ED, emergency department; ICU, intensive care unit; GCS, Glasgow coma scale.

**Supplementary Table 7.**

**Subgroup analysis by shock status comparing primary outcomes (in-hospital mortality) between early intubation and delayed intubation**

|  | **With vasopressor** | | **Without vasopressor** | |
| --- | --- | --- | --- | --- |
|  | **OR (95% CI)** | **P-value** | **OR (95% CI)** | **P-value** |
| Delayed intubation | Reference group |  | Reference group |  |
| Early intubation | 0.72 (0.47-1.12) | 0.12 | 0.60 (0.18–1.96) | 0.49 |
| Age | 1.02 (1.01–1.04) | 0.0006 | 1.01 (0.98–1.04) | 0.38 |
| SOFA score | 1.22 (1.15–1.28) | <0.0001 | 1.34 (1.14–1.58) | 0.0003 |
| Non-frail (CFS 1-4) | Reference group |  | Reference group |  |
| Frailty (CFS 5-8) | 1.69 (1.13–2.52) | 0.021 | 2.54 (0.82–7.92) | 0.097 |
| Unknown frailty | 1.53 (1.00–2.34) | 0.057 | 2.76 (0.82–9.26) | 0.097 |
| Hospital admission source |  |  |  |  |
| other hospital (without ED) | 0.80 (0.45–1.45) | 0.46 | 0.33 (0.04–2.79) | 0.27 |
| other healthcare facilities | 0.42 (0.13–1.29) | 0.12 | NA | 0.99 |
| rehabilitation | NA | 0.99 | NA | NA |
| other hospital-ED | 0.82 (0.48–1.39) | 0.50 | 0.59 (0.14–2.43) | 0.40 |
| Treatment limitations at ICU admission | 2.36 (1.48–3.76) | 0.0003 | 2.81 (0.86–9.14) | 0.10 |
| Emergency response admission | 1.48 (1.10–2.00) | 0.010 | 0.64 (0.26–1.54) | 0.36 |

OR, adjusted odds ratios; CI, confidence intervals; SOFA, Sequential Organ Failure Assessment; CFS, clinical frailty scale; ED, emergency department; ICU, intensive care unit; GCS, Glasgow coma scale.

**Supplementary Table 8.**

**Subgroup analysis by frailty status comparing primary outcomes (in-hospital mortality) between early intubation and delayed intubation**

|  | **Frail** | | **Non frail** | | **Unknown frail** | |
| --- | --- | --- | --- | --- | --- | --- |
|  | **OR (95% CI)** | **P-value** | **OR (95% CI)** | **P-value** | **OR (95% CI)** | **P-value** |
| Delayed intubation | Reference group |  | Reference group |  | Reference group |  |
| Early intubation | 1.01 (0.52–1.99) | 0.96 | 0.55 (0.29–1.03) | 0.062 | 0.78 (0.34–1.80) | 0.46 |
| Age | 1.02 (1.00-1.04) | 0.13 | 1.03 (1.01–1.05) | 0.0037 | 1.02 (0.99–1.04) | 0.26 |
| SOFA score | 1.19 (1.09–1.30) | 0.0005 | 1.23 (1.14–1.33) | <0.0001 | 1.30 (1.18–1.44) | <0.0001 |
| Non-frail (CFS 1-4) | NA |  | NA |  | NA |  |
| Frailty (CFS 5-8) | NA |  | NA |  | NA |  |
| Unknown frailty | NA |  | NA |  | NA |  |
| Hospital admission source |  |  |  |  |  |  |
| other hospital (without ED) | 0.89 (0.32–2.49) | 0.80 | 1.28 (0.51-3.18) | 0.60 | 0.61 (0.25–1.46) | 0.31 |
| other healthcare facilities | 0.29 (0.08–1.07) | 0.054 | NA | 0.99 | 1.02 (0.10–10.57) | 0.84 |
| rehabilitation | NA | 0.99 | NA | 1.00 | NA | 0.99 |
| other hospital-ED | 0.94 (0.42–2.15) | 0.92 | 0.75 (0.35–1.61) | 0.51 | 0.85 (0.30–2.38) | 0.64 |
| Treatment limitations at ICU admission | 2.46 (1.32–4.61) | 0.0039 | 2.68 (1.22–5.89) | 0.017 | 2.40 (0.97–5.97) | 0.044 |
| Emergency response admission | 1.83 (1.07–3.11) | 0.039 | 1.08 (0.66–1.77) | 0.69 | 1.38 (0.84–2.27) | 0.19 |

OR, adjusted odds ratios; CI, confidence intervals; SOFA, Sequential Organ Failure Assessment; CFS, clinical frailty scale; ED, emergency department; ICU, intensive care unit; GCS, Glasgow coma scale.

**Supplementary Table 9.**

**Comparison of secondary outcomes (mortality at three, six and twelve months of follow-up) between early intubation and delayed intubation with multivariable logistic regression analysis**

| **Secondary Outcomes** | **3-month mortality** | | **6-month mortality** | | **12-month mortality** | |
| --- | --- | --- | --- | --- | --- | --- |
|  | **OR (95% CI)** | **P-value** | **OR (95% CI)** | **P-value** | **OR (95% CI)** | **P-value** |
| Delayed intubation | Reference group |  | Reference group |  | Reference group |  |
| Early intubation | 0.66 (0.46-0.97) | 0.038 | 0.76 (0.53-1.10) | 0.16 | 0.75 (0.53-1.06) | 0.11 |
| Age | 1.03 (1.02 -1.04) | <0.001 | 1.03 (1.02-1.04) | <0.001 | 1.03 (1.02-1.04) | <0.001 |
| SOFA score | 1.18 (1.12-1.24) | <0.001 | 1.15 (1.10-1.20) | <0.001 | 1.14 (1.09-1.19) | <0.001 |
| Non-frail (CFS 1-4) | Reference group |  | Reference group |  | Reference group |  |
| Frail (CFS 5-8) | 2.12 (1.47-3.05) | <0.001 | 2.47 (1.75-3.49) | <0.001 | 2.50 (1.79-3.49) | <0.001 |
| Unknown frailty | 1.70 (1.16-2.48) | 0.006 | 1.66 (1.15-2.39) | 0.007 | 1.57 (1.11-2.23) | 0.012 |
| Hospital admission source |  |  |  |  |  |  |
| other hospital (without ED) | 1.22 (0.75-1.98) | 0.42 | 1.32 (0.83-2.09) | 0.24 | 1.19 (0.76-1.87) | 0.46 |
| other healthcare facilities | 0.64 (0.26-1.57) | 0.32 | 0.58 (0.24-1.36) | 0.21 | 0.49 (0.21-1.16) | 0.10 |
| rehabilitation | NA | 0.99 | 0.55 (0.06-5.50) | 0.60 | 0.46 (0.05-4.56) | 0.51 |
| other hospital-ED | 1.05 (0.66-1.65) | 0.85 | 1.11 (0.72-1.71) | 0.63 | 1.11 (0.74-1.68) | 0.60 |
| Treatment limitations at ICU admission | 2.25 (1.50-3.38) | 0.001 | 2.26 (1.52-3.35) | <0.001 | 2.14 (1.45-3.15) | 0.001 |
| Emergency response admission | 1.28 (0.97-1.69) | 0.09 | 1.31 (1.00-1.72) | 0.06 | 1.31 (1.01-1.70) | 0.05 |

OR, adjusted odds ratios; CI, confidence intervals; SOFA, Sequential Organ Failure Assessment; CFS, clinical frailty scale; ED, emergency department; ICU, intensive care unit; GCS, Glasgow coma scale.

**Supplementary Table 10.**

**Comparison of secondary outcomes (ICU length of stay) between early intubation and delayed intubation with multivariable linear regression analysis**

|  | **ICU Length of stay** | |
| --- | --- | --- |
|  | **Point Estimate**  **(95% CI)** | **P-value** |
| Delayed intubation | Reference group |  |
| Early intubation | -70.60 (-93.71 to -47.50) | <0.001 |
| Age | -0.10 (-0.69 to 0.50) | 0.69 |
| SOFA score | 6.69 (3.87 to 9.51) | <0.001 |
| Non-frail (CFS 1-4) | Reference group |  |
| Frail (CFS 5-8) | -8.18 (-29.99 to 13.64) | <0.001 |
| Unknown frailty | -18.91 (-43.87 to 6.05) | 0.006 |
| Hospital admission source |  |  |
| other hospital (without ED) | 2.37 (-19.73 to 40.47) | 0.50 |
| other healthcare facilities | 3.95 (-63.14 to 43.25) | 0.71 |
| rehabilitation | 5.99 (-78.06 to 200.05) | 0.42 |
| other hospital-ED | 98.07 (-8.22 to 46.36) | 0.17 |
| Treatment limitations at ICU admission | -48.40 (-76.89 to -19.92) | 0.001 |
| Emergency response admission | 2.67 (-16.08 to 21.43) | 0.86 |

CI, confidence intervals; SOFA, Sequential Organ Failure Assessment; CFS, clinical frailty scale; ED, emergency department; ICU, intensive care unit.

**Supplementary Table 11.**

**Comparison of secondary outcomes (hospital length of stay) between early intubation and delayed intubation with multivariable linear regression analysis**

|  | **Hospital Length of stay** | |
| --- | --- | --- |
|  | **Point Estimate**  **(95% CI)** | **P-value** |
| Delayed intubation | Reference group |  |
| Early intubation | -3.05 (-6.89 to 0.80) | 0.07 |
| Age | 0.04 (-0.06 to 0.14) | 0.38 |
| SOFA score | -0.04 (-0.51 to 0.43) | 0.17 |
| Non-frail (CFS 1-4) | Reference group |  |
| Frail (CFS 5-8) | -0.24 (-3.90 to 3.42) | 0.60 |
| Unknown frailty | -1.69 (-5.69 to 2.31) | 0.38 |
| Hospital admission source |  |  |
| other hospital (without ED) | 2 (-5.64 to 4.40) | 0.87 |
| other healthcare facilities | 6 (-6.49 to 11.42) | 0.61 |
| rehabilitation | 8 (10.97 to 57.78) | 0.007 |
| other hospital-ED | 8 (-6.73 to 2.37) | 0.44 |
| Treatment limitations at ICU admission | -4.71 (-9.50 to 0.09) | 0.06 |
| Emergency response admission | 4.29 (1.22 to 7.37) | 0.007 |

CI, confidence intervals; SOFA, Sequential Organ Failure Assessment; CFS, clinical frailty scale; ED, emergency department; ICU, intensive care unit.
